# When guilt works: a comprehensive meta-analysis of guilt appeals

**DOI:** 10.3389/fpsyg.2023.1201631

**Published:** 2023-09-28

**Authors:** Wei Peng, Qian Huang, Bingjing Mao, Di Lun, Ekaterina Malova, Jazmyne V. Simmons, Nick Carcioppolo

**Affiliations:** ^1^Edward R. Murrow College of Communication, Washington State University, Pullman, WA, United States; ^2^Department of Communication, University of North Dakota, Grand Forks, ND, United States; ^3^TSET Health Promotion and Research Center, Stephenson Cancer Center, University of Oklahoma Health Sciences Center, Oklahoma, OK, United States; ^4^Department of Health Disparities Research, University of Texas MD Anderson Cancer Center, Houston, TX, United States; ^5^Simon Business School, University of Rochester, Rochester, NY, United States; ^6^Division of Health Science, School of Allied Health Sciences, Florida Agricultural and Mechanical University, Tallahassee, FL, United States; ^7^School of Communication, University of Miami, Coral Gables, FL, United States

**Keywords:** guilt, emotion, persuasion, attitudes, behaviors, meta-analysis

## Abstract

**Introduction:**

Guilt appeals are widely used as a persuasive approach in various areas of practice. However, the strength and direction of the persuasive effects of guilt appeals are mixed, which could be influenced by theoretical and methodological factors.

**Method:**

The present study is a comprehensive meta-analysis of 26 studies using a random-effects model to assess the persuasive effects of guilt appeals. In total, 127 effect sizes from seven types of persuasive outcomes (i.e., guilt, attitude, behavior, behavioral intention, non-guilt emotions, motivation, and cognition) were calculated based on 7,512 participants.

**Results:**

The analysis showed a small effect size of guilt appeals [*g* = 0.19, 95% CI (0.10, 0.28)]. The effect of guilt appeals was moderated by the theoretical factors related to appraisal and coping of guilt arousal, including attributed responsibility, controllability and stability of the causal factors, the proximity of perceiver-victim relationship, recommendation of reparative behaviors, and different outcome types. The effect was also associated with methods used in different studies.

**Discussion:**

Overall, the findings demonstrated the persuasive effects of guilt appeals, but theoretical and methodological factors should be considered in the design and testing of guilt appeals. We also discussed the practical implications of the findings.

## Introduction

Guilt appeals are a type of persuasive communication that evokes feelings of guilt to influence persuasive outcomes (O'Keefe, 2000). In essence, guilt appeals arouse guilt by activating a self-evaluative process through which the perceiver finds their behavior deviating from their moral and social standards (Tangney et al., 2007; Greenbaum et al., 2020). Aroused guilt elicits reparative behaviors toward the victim, such as apologies, compensation, and cooperative behaviors, to undo the harm and reduce conflicts (de Hooge et al., 2011; Chrdileli and Kasser, 2018). For this reason, guilt has been used to promote compliance in the forms of advertisements, health or educational materials, electronic information, and interpersonal conversations (Antonetti et al., 2018; Xu and Guo, 2018).

Empirical research testing the effects of guilt appeals has shown mixed results (Shehu et al., 2013; Noble et al., 2014; Antonetti et al., 2018). The inconsistent findings draw attention to several prominent issues in guilt appeal research. First, guilt induction involves multiple processes, meaning that the antecedents of guilt arousal may vary across different studies (Tangney et al., 2007; Greenbaum et al., 2020). One factor is the attribution of the cause of other's suffering (Tracy and Robins, 2006). Previous studies elicited guilt by focusing on the victim's suffering only (Chang, 2012; Lee, 2013) or by additionally attributing the responsibility to the perceiver (Antonetti et al., 2018). In addition, the relational distance between the perceiver and the victim could affect guilty feelings (Ghorbani et al., 2013). Second, guilt appeals seek to motivate reparative behaviors, but including such information often makes the perceiver feel coerced, resulting in weaker effectiveness (Graton et al., 2016). Third, a survey of studies testing guilt appeals has found a mixture of research methods and interests that vary in topic contexts, induction approaches, and methods of measurement.

Overall, distinct theoretical and methodological factors from past studies increase the uncertainty of guilt appeal research, calling for meta-analytical studies. Previous meta-analyses have demonstrated the effects of guilt appeals (O'Keefe, 2000; Boster et al., 2016; Xu and Guo, 2018; Turner and Rains, 2021) but had several limitations. Not all meta-analyses systematically accounted for various processes involved in guilt appeals under a consistent theoretical framework. Abbreviated investigations of non-theoretical moderators have been conducted so far. As a result, the variations of guilt induction and coping were not examined. Moreover, heterogeneity in methodological approaches, including but not limited to contexts, induction methods, and measurement in extant studies on guilt appeals, had not been fully evaluated.

**Figure 1:**
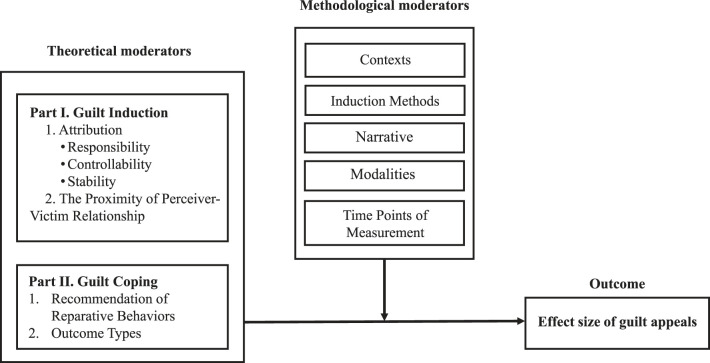
The conceptual model of the meta-analysis of guilt appeals with theoretical and methodological moderators.

Therefore, the present meta-analysis aimed to achieve three goals. First, this study sampled a larger body of guilt appeal studies to investigate the overall effect size. Second, the study centered on the emotional mechanism of guilt appeals to evaluate relevant theoretical moderators that had not been examined before. Third, it investigated the moderating effects of methodological differences. The following section is organized into two parts. The first part examines the theoretical framework and mechanism of guilt appeals, which guides the review of theoretical moderators related to the processes of guilt induction and coping. The second part is a review of key methodological moderators—contexts, induction methods, narratives, modalities, and time points of measurement. A conceptual model of the present meta-analysis is available in Figure 1.

## Theoretical framework and moderators of guilt appeals

Guilt appeals arouse guilt as a discrete emotion to persuade (O'Keefe, 2000). There is substantial agreement that human emotions are adaptive responses of an organism to stimuli in the external environment (Mulligan and Scherer, 2012; Roseman, 2013). Each discrete emotion is aroused by a unique pattern of appraisals that evaluates the external stimulus with regard to different dimensions, such as motivational relevance, goal congruence, expectedness, control, and agency (Smith and Ellsworth, 1985; Moors et al., 2013; Moors, 2020). The perceiver also appraises the coping ability to reduce the demands from emotions arising from this event (Roseman, 2013; Scherer and Moors, 2019). This coping appraisal prompts mental and behavioral strategies to meet the needs of the personal goal and regulate the aroused emotions (Roseman, 2013; Scherer and Moors, 2019). Because the adaptive responses of emotions can change motivational priorities to address the causal factors, theorists reason that emotions can cause biased estimation of the possibility and desirability of an object, leading to changes in decision-making processes (Nabi, 2003; DeSteno et al., 2004; So et al., 2015).

Based on the appraisal theories, guilt appeals should be designed with two pivotal components: guilt induction and coping (Nabi, 1999; O'Keefe, 2002). In the first step, guilt appeals induce guilt in the perceiver by guiding their cognitive appraisals to evaluate specific dimensions of the stimulus event (O'Keefe, 2002). Once feeling guilty, a perceiver evaluates and adopts possible strategies to control the emotion (Dearing et al., 2005; de Hooge et al., 2011; Behrendt and Ben-Ari, 2012). Thus, in the second step, guilt appeals can recommend specific actions of the perceiver to regulate guilty feelings (O'Keefe, 2002). Based on the framework of discrete emotions, variations in theoretical factors related to guilt induction and coping can influence the effects of guilt appeals.

### Guilt induction as theoretical moderators

Guilt induction is the first component of guilt appeals. Theoretical models conceptualize guilt as a self-conscious emotion elicited by the inconsistency between one's self-representation and moral or social standards (Tracy and Robins, 2006; Tangney et al., 2007). Guilt is associated with complex concepts of self and socialization needs, such as maintaining a friendship, as opposed to survival instincts (de Hooge et al., 2007; Cryder et al., 2012). We review several key factors of guilt induction, including attribution dimensions associated with responsibility, controllability, stability, and the proximity of the perceiver–victim relationship (Tracy and Robins, 2006; Tangney et al., 2007; Ghorbani et al., 2013).

#### Attribution

Attribution is an essential part of the appraisal in guilt elicitation (Weiner et al., 1982). In the attribution process, the perceiver evaluates the cause of the victim's suffering presented in guilt appeals regarding its responsibility, controllability, and stability (Tracy and Robins, 2004). From the perspective of the persuader, how the cause is presented and perceived concerning these three attribution dimensions is critical to eliciting guilt. The first dimension of attribution is responsibility. Emotion research shows that guilt arises from the sense of responsibility for inflicting harm on others (Tangney et al., 2007; Roberts et al., 2014; Miceli and Castelfranchi, 2018). Responsibility plays an essential role in the social and moral function of guilt as it motivates people to acknowledge their mistakes and take reparative actions in the aftermath of transgressions (Tangney et al., 2007). To activate the appraisal of responsibility, guilt appeals blame the perceiver as the cause of others' suffering in a specific social or moral context (Harth et al., 2013; Antonetti et al., 2018).

However, responsibility is not always a necessary condition of guilt arousal. Existential guilt is associated with a person's broader perception of their meaning and expectations in a human society (Binder, 2022). This type of guilt is often evoked by moral responsibility or social obligation, which does not result from an actual transgression one commits but from one's perceived failure to fulfill their meaning and duties in society (Zimmermann et al., 2011). Thus, existential guilt extends beyond mere compensation and punishment as described by Tangney et al. (2007) and could motivate people to engage in broader issues. Others contend that existential guilt is possibly driven by empathy, which links one with others in a social relation, to reduce others' suffering (Baumeister et al., 1994; Roberts et al., 2014). Thus, people would feel guilty about inequality impacting others, even though they did not cause its occurrence (O'Connor et al., 2000; Harth et al., 2013; Dull et al., 2021). For example, guilty feelings are experienced by the survivors of natural disasters (Hugelius et al., 2017; Dang et al., 2022), workplace layoffs (Karfakis and Kokkinidis, 2018; Mujtaba and Senathip, 2020), or cancer (Perloff et al., 2019). For this reason, without assigning responsibility, guilt appeals can still be persuasive by evoking the perceivers' role in a social system and their behaviors to address broad social issues (e.g., climate change, equality, and poverty) (Chang, 2011).

The other two attributional dimensions are stability and controllability (Tangney et al., 2007). The stability dimension is crucial to distinguish guilt from other moral emotions, such as shame (Tracy and Robins, 2004). Compared with shame, guilt arises from one's attention to the unstable aspect of the self, such as behaviors, as opposed to one's personality, character, and other stable aspects (Greenbaum et al., 2020). To influence the appraisal of stability, guilt appeals provide evaluation information showing that the nature of a causal event is changeable (Xu and Guo, 2018). The attribution to unstable causes is often achieved by focusing on a specific incident or behavior of the perceiver as the cause of the victim's suffering (Roberts et al., 2014).

Moreover, to influence the appraisal of controllability, guilt appeals attribute the victim's suffering to a cause over which the perceiver has control (Antonetti and Maklan, 2014). As a result, the perceiver can develop a stronger sense of agency over the process and consequences of the causal event (Roberts et al., 2014). A theoretical review explains that the action taken by the self is an integral part of morality, which is linked to guilt as a moral emotion (Jennings et al., 2015), that is, in addition to the person's identity and environment, the actions a person takes to influence society and self-shape the characteristics of their morality (Jennings et al., 2015). On the other hand, controllability should be distinguished from stability attribution in the appraisal of guilt (Tracy and Robins, 2004). While a cause is attributed to be unstable, a person might not feel guilty if the locus of control is external (Antonetti and Maklan, 2014). Thus, the effects of guilt appeals might depend on whether the guilt appeals attribute a negative event to a cause that is controlled by the perceiver.

#### The proximity of perceiver–victim relationships

The elicitation of guilt can vary by the proximity of the relationships between the perceiver and the victim (Baumeister et al., 1994). Transgressors usually have a stronger feeling of guilt when they are interpersonally close to the victims, such as friends or family (Ghorbani et al., 2013). Because guilt involves one's self-representation in a moral or social context, people can more easily appraise the effects of their misdeeds on others and empathetically feel their suffering if there is a close connection (Tangney et al., 2007; Ruckstaetter et al., 2017). In addition, transgressors can be motivated to reconcile their transgressions due to the concern about alienating those in close relationships (Ghorbani et al., 2013).

Additionally, a personal relationship is not always required for guilt induction. Guilt can be elicited by the wrongdoings committed by in-group members onto others (Iyer et al., 2007; Zimmermann et al., 2011). For example, people felt guilty about their country's actions toward disadvantaged groups in the Dutch colonization of Indonesia (Doosje et al., 1998) and the Israeli–Palestinian conflicts (Roccas et al., 2006). In response to the finding, Doosje et al. (1998) explained that transgressions committed by in-group members might threaten the perceiver's social identity and arouse collective guilt. In addition, individuals may develop a sense of community toward workplaces or other institutions (Nowell and Boyd, 2014). Guilty feelings might result from the perception that one had not responded to the misfortune of their community (Zimmermann et al., 2011).

Thus, we seek to understand whether the proximity of the relationship between a perceiver and a depicted victim moderates the effectiveness of guilt appeals. When appraising a negative event, a perceiver may react differently after assessing whether the victim is in a close relationship. Hence, the proximity of the perceiver–victim relationship will be tested as a theoretical moderator.

### Guilt coping as a theoretical moderator

There is a clear consensus among emotion theories that negative human emotions must be regulated by changing the causal event directly or releasing the mental state from its influence (Lazarus, 1991; Ellsworth and Scherer, 2003; Roseman, 2013). Once feeling guilty, the perceiver seeks and evaluates possible strategies to cope with the emotion (Tracy and Robins, 2006; Tangney et al., 2007). In this process, following the guilt induction process, guilt appeals provide the perceiver with a certain action or counterfactual thinking to remedy guilt-arousing causes (O'Keefe, 2002; Antonetti et al., 2015). Thus, the meta-analysis will test the following two moderators to understand how the effects of guilt appeals are associated with the regulation of aroused guilt.

#### Recommendation of reparative behaviors

Guilt appeals in various contexts provide immediately accessible information to help the perceiver take measures directly against the source of the negative feelings (Agrawal and Duhachek, 2010; Antonetti et al., 2015). Extensive research has shown that guilt motivates reparative behaviors to amend wrongdoings and undo the harm to others (Ketelaar and Tung Au, 2003; Tangney et al., 2007; Shen, 2018; Vaish, 2018). The reparation process helps transgressors control their guilty feelings (Tangney et al., 2007). Confession, apology, cooperative, and prosocial behaviors are among the most common reparative behaviors in social interactions (Tangney et al., 2007; de Hooge et al., 2011; Behrendt and Ben-Ari, 2012). For example, Ilies et al. (2013) found that in the workplace, employees who felt guilty about their counterproductive work behaviors were more likely to offer helping behaviors. In interpersonal conflicts, guilty feelings prompt people to engage in cooperative approaches (Behrendt and Ben-Ari, 2012). Other studies suggest that apologies were related to guilt, but not to shame (Ruckstaetter et al., 2017; Chrdileli and Kasser, 2018).

For this reason, guilt appeals might be more persuasive if recommending reparative actions can immediately satisfy the perceiver's need to make amends (Graton and Mailliez, 2019). In addition, guilt appeals with reparation suggestions convey the repairable nature of the events for which people feel guilty (Tangney et al., 2007), that is, the negative consequences of someone's wrongdoings can be counteracted by the recommended actions in guilt appeals (Graton et al., 2016). If no actions or remedies are provided in guilt appeals, the perceiver might feel that the negative consequences cannot be offset, and their recovery from guilt is inhibited (LeBlanc et al., 2020). In turn, people would seek to deal with the negative feelings directly without changing the cause (Antonetti and Baines, 2015). As a result, guilt appeals may be avoided or rejected (Graton et al., 2016).

However, explicit information about reparative behaviors does not necessarily strengthen the effectiveness of guilt appeals. In a series of experiments, Graton and Ric (2017) found that guilty feelings have increased attention to and positive attitudes toward implicit cues of making amends. Additional demands for reparative behaviors, such as asking for charitable donations, could make people feel coerced. These findings bring uncertainty into whether recommending reparative behaviors in guilt appeals can influence persuasive effectiveness.

#### Outcomes as a theoretical moderator

The process of guilt appeals may vary outcomes, resulting in variations in the effect size of guilt appeals. The first outcome is guilt, which often serves as a validity indicator of guilt appeals (O'Keefe, 2000). Guilt is a mediating process of guilt appeals before more distal persuasion outcomes. Through the elicitation of guilt, guilt appeals engender persuasion resulting from the perceiver's motivation to control the guilty feelings (Tangney et al., 2007; Allard and White, 2015). Guilt elicited by guilt appeals is thus expected to be consistent and strong. However, if the stimulus event framed in a guilt appeal is relatively more important to the perceiver's goals other than maintaining the social or moral dimension of self-identity, more ambiguous or other emotions may arise, reducing the intensity of guilty feelings (Tracy and Robins, 2006; Tangney et al., 2007).

Attitude is another essential outcome of guilt appeals. Attitude is generated from an appraisal process in which the perceiver evaluates the attributes of the persuasion object (Fishbein and Ajzen, 1975). While early theoretical work primarily focused on cognitive antecedents of attitudinal assessments, more recent studies show that emotional information is an essential dimension of the evaluations for attitude (Fishbein and Middlestadt, 1995; Zanna and Rempel, 2008). Guilt appeals influence attitudes by providing information that can cause biased estimation of the value and probability of specific attributes associated with a persuasion object (DeSteno et al., 2004). Variations of attitude depend on whether certain attributes of the object are appraised as potentially helpful to cope with the aroused guilty feelings and attain one's goal related to social and moral issues (Allen et al., 2005; Antonetti and Baines, 2015). The desirability of the persuasion object can also be lower if the guilt appeals elicit other emotions or conflict with incidental emotions (DeSteno et al., 2004).

Behaviors and behavioral intentions are considered focal outcomes of guilt appeals. Behavior is at the center of guilt experiences. Guilt is elicited by specific wrongdoings and the regulation process is often achieved by reparative behaviors (Tangney et al., 2007; Roberts et al., 2014). As an emotion regulation mechanism, behavioral actions are taken by individuals to address the problems that cause emotions (Scherer and Moors, 2019). In this sense, guilt appeals suggest specific behavioral actions that can undo the harm to the victim and reduce guilty feelings (Tangney et al., 2007; Antonetti and Baines, 2015). Thus, guilt appeals are often used in persuasion to gain behavioral compliance (Agrawal and Duhachek, 2010; Chang, 2012). If behavioral outcomes provide little help to control negative feelings, maladaptive guilt experiences can result in rejection or avoidance of the entire message and recommended behaviors (Kim et al., 2011; Antonetti and Baines, 2015).

Lastly, guilt is expected to change motivation and cognition (Bradley et al., 2001; Pessoa, 2009). Aroused guilt disrupts thoughts and directs cognitive resources to fix eliciting events and control aversive feelings (Tangney et al., 2007; Scherer and Moors, 2019). Motivation is an intermediate process between emotional arousal and cognitive or behavioral responses. Emotion is aroused when a person's motivation-related goal is satisfied or at risk (Roseman, 2013). In response, motivation prioritizes specific cognitive resources (e.g., attention) and executive functions (e.g., behaviors) to act toward the goal (Pessoa, 2009). Thus, guilt can motivate a person to engage in reparation behaviors that help maintain the goal related to one's self-representation or interpersonal relationships (Tangney et al., 2007; Greenbaum et al., 2020).

## Methodological moderators of guilt appeals

Methodological differences should be assessed to understand the varying effects of guilt appeals. The effects of guilt appeals can be influenced by different contexts (Massi Lindsey, 2005; Chang, 2012), induction methods (e.g., Allard and White, 2015; La Ferle et al., 2019), or outcome variables (Noble et al., 2014). Below, we review methodological factors related to the effectiveness of guilt appeals.

### Contexts of persuasion objects

Different contexts of persuasion objects can account for variations in effects in that each context may result in different appraisal patterns (e.g., causal attribution, reparative behaviors, and personal relevance). Commonly seen in advertising and marketing, guilt appeals are used to promote a product or service by provoking the perceiver's examination of self-representation against a person's goal and claiming how the persuasion object (e.g., a product) helps them reach the goal (Antonetti and Baines, 2015). Guilt appeals can also change self-efficacy and behaviors in health and medical contexts (Xu and Guo, 2018). Guilt appeals in a health-related context seek to portray certain health behaviors as wrongdoing that violates socially acceptable rules and generates negative impacts on people in social relationships, such as the harmful effects of human papillomavirus infection on a sexual partner (Babin, 2009). Another important context is social issues with broad impacts, such as environmental protection (Baek and Yoon, 2017; Schneider et al., 2017) and charity giving (Chang, 2011; Xu, 2022). Guilt feelings arise when people consider themselves not fulfilling their moral and social responsibility and these causes (Zimmermann et al., 2011; Urbonavicius et al., 2019). Guilt appeals in this context motivate prosocial and altruistic behaviors to alleviate guilty feelings (de Hooge et al., 2011; Urbonavicius et al., 2019).

### Induction methods

Two methods of guilt induction commonly used in previous research could influence the effects of guilt appeals. One method is based on persuasive messages, which provide a brief piece of information that matches the core appraisal theme of guilt to arouse guilty feelings in the perceiver (Nabi, 1999, 2003). For instance, a guilt appeal message portrays the victim's suffering and frames the perceiver's controllable behavior as the cause. The other method uses recall and imagination, commonly through verbal instructions in interpersonal communications (Tracy and Robins, 2006). The perceiver is asked by the persuader to recall a salient incident in their lives that once aroused guilt and then to re-experience it in the current setting (Allard and White, 2015; Graton et al., 2016).

The influence of induction methods on guilt appeals might center on the relevance of information. According to the self-reference effect, information about oneself is clearly organized in memory and easily accessible (Block, 2005). Despite its popularity, message-based guilt appeals can be perceived as less relevant to the perceiver when describing the suffering of an unknown party (Ghorbani et al., 2013). As a result, the effects of guilt appeals may be hampered (Chang, 2012; Ghorbani et al., 2013). In this sense, recall might be advantageous to elicit self-conscious emotions like guilt (Tracy and Robins, 2006; Siedlecka and Denson, 2019). Through recall, the perceiver can reproduce the emotional experience and examine whether certain aspects of themselves have caused negative impacts on others (Tracy and Robins, 2006). Thus, this study will test whether induction methods moderate the effects of guilt appeals.

### Narrative

A growing body of literature points to emotional experiences as the foundation of narrative persuasion (Green and Brock, 2000; Nabi and Green, 2015; Liu and Yang, 2020). Using narrative in guilt appeals has also been tested in the literature. Past studies have narratives to evoke feelings of guilt by providing the victim's stories or by engaging the perceiver in summoning personal experiences (Graton et al., 2016; Antonetti et al., 2018, study 1b). In contrast, non-narrative guilt appeals typically use reasoning, logical arguments, and statistical information (Chang, 2012; Antonetti et al., 2018, study 1a). However, the overall effects of narrative vs. non-narrative persuasion are mixed (Shen et al., 2015; Freling et al., 2020). We seek to test whether using narratives in guilt appeals is associated with stronger persuasiveness.

### Modalities

Guilt appeals can benefit from different modalities (e.g., text, audio, video, or mixed modes), but the effects may vary. According to multimedia learning theory (Mayer and Moreno, 2003; Mayer, 2014), information in multiple modalities increases attention to essential content, adds visual cues to exemplify the central theme, and reduces the cognitive burden through redundant information. Visual content may build mental experiences through which a person can experience the if-then scenario in guilt arousal (Chien and Chang, 2012; Antonetti et al., 2015). Thus, it is reasonable to argue that these benefits of multimedia information could support the processing of guilt appeals to achieve the intended effects. However, multimedia information with a poor design might also distract a perceiver from scrutinizing the claims in guilt appeals (Mayer, 2014). For example, irrelevant visual information might cause incidental processing and unintended effects (Sung and Mayer, 2012). Given limited evidence to inform the most effective modality for guilt appeal, the present meta-analysis tests whether the effectiveness of guilt appeals varies by different modalities.

### Time points of measurement

The effects of guilt appeals may depend on different time points of measurement after persuasion occurs. Most existing studies measured the effects of guilt appeals immediately after exposure (Peloza et al., 2013; Graton et al., 2016) because emotional responses are often transient (Brosch, 2021; Ogunfowora et al., 2023). Nevertheless, recent research suggests that immediate measurement might not capture the desired effects of guilt appeals as people might delay their decisions to adopt the suggested behavior in persuasion (Antonetti et al., 2018; Brosch, 2021; Kemp, 2023). Thus, the present meta-analysis will understand how different measurement time points can cause variation in the observed persuasive effects of guilt appeals.

## The present meta-analysis

The persuasive effects of guilt appeals have been synthesized by past meta-analyses, but several gaps remain to be addressed. Both O'Keefe (2000, p. 74) and Boster et al. (2016) included guilt appeal studies based on the transgression-compliance mechanism and found medium effect sizes (*r*s = 0.28 and 0.26, respectively). However, both studies presumed based on *a priori* knowledge that guilt would drive behavioral compliance of transgressors. Thus, their findings should not be interpretable as the effects of guilt appeal because transgressors' behavioral compliance is not necessarily explained by guilt (Cialdini et al., 1973; Baumeister et al., 1994). The other three meta-analyses studied guilt appeals that intentionally aroused guilt emotion for persuasive outcomes but suffered from multiple limitations. First, O'Keefe (2000, p. 80) compared five guilt appeal studies with varying explicitness and found that more explicit guilt appeals were associated with less persuasive effects (*r* = −0.26). However, this meta-analysis did not explicate the processes related to the intensity of guilt induction. Second, Xu and Guo (2018) demonstrated moderate effects of guilt-based persuasive messages but their findings were limited to health-related outcomes. In addition, several included studies manipulated persuasive constructs that were not intended to evoke guilt (e.g., norm perception, Lee and Paek, 2013). Furthermore, their analysis only examined a limited number of moderators, including message modality, self/other focus, and health behaviors. In a more recent meta-analysis, Turner and Rains (2021) found a small effect size of guilt appeals on attitudes or behavioral intentions and medium effects on guilt and anger. However, similar to previous meta-analyses, moderators associated with the appraisal dimensions of guilt arousal, coping process, and outcomes were not of primary interest. Their study did not account for heterogeneity in outcomes of guilt appeals.

The present study hence aimed to conduct a comprehensive synthesis of guilt appeals. There are four strengths of this study. First, we examined the studies that intentionally evoked guilt as an emotional mechanism of persuasion. Persuasion studies that manipulated the constructs not intended to evoke guilt were excluded. Thus, this meta-analysis provided a more robust and accurate estimate of the effectiveness of guilt appeals. Second, guided by appraisal theories of emotion, we systematically accounted for key factors associated with guilt induction and coping. The absence of such inquiries in previous studies could contribute to inconsistent results (O'Keefe, 2002; Tangney and Dearing, 2002). Thus, examining these factors would offer important insights into variations in guilt arousal and persuasive outcomes. Third, previous meta-analyses primarily assessed the effects on attitude and behaviors. In contrast, our study evaluated additional dependent variables to assess the overall effects of guilt appeals thoroughly. Fourth, we evaluated various methodological characteristics of past guilt appeal studies. As the body of guilt appeals expands, this analytic approach can critically address the limitation of previous meta-analyses in which methodological differences were not of focus.

The first goal of this meta-analysis is to understand the overall effectiveness of guilt appeals. To this end, we proposed the following research question.

RQ1: Compared with equivalent neutral true control conditions, do guilt appeals generate greater persuasive effects?

Also, as reviewed, theoretical factors involved in guilt induction and coping could influence the effectiveness of guilt appeals. The following hypotheses were proposed to test whether these factors moderated the effects of guilt appeals.

H1: The effects of guilt appeals are moderated by the presence of attribution dimensions related to (a) responsibility, (b) controllability, or (c) stability in guilt induction.

H2: The effects of guilt appeals are moderated by the proximity of the relationship between the perceiver and the victim in guilt induction.

H3: The effects of guilt appeals are moderated by the recommendation of reparative behaviors to cope with aroused guilt.

H4: The effects of guilt appeals are moderated by different types of outcomes.

Across a large body of past research, various method approaches to guilt appeals may influence the effectiveness of guilt appeals. The following research question is proposed to understand which methodological factors moderate the effects of guilt appeals.

RQ2: Are the effects of guilt appeals moderated by (a) context of persuasion objects, (b) induction methods, (c) narrative, (d) modalities, and (e) time point of measurement?

## Methods

### Search process

We used the Population, Intervention, Comparator, Outcomes, and Study (PICOS) design model in accordance with the PRISMA guideline (Moher et al., 2009) to develop the search strategy. The details of our PICOS criteria are provided in Table 1.

**Table 1:** The PICOS (populations, interventions, comparators, outcomes, study design) inclusion criteria and initial article screening results.

**Category**	**Inclusion**	**First round of screening^a^**	**Second round of screening^b^**
Populations	The general population. No demographic group is excluded.	None was excluded due to this criterion.	None was excluded due to this criterion.
Interventions	Any persuasion that manipulates guilt induction to change participants' psychological outcomes (e.g., beliefs, attitudes, emotions) and/or behaviors. Exclude: 1) Persuasions that do not manipulate guilt induction to change participants' psychological outcomes (e.g., beliefs, attitudes, emotions) and/or behaviors. 2) Persuasions that manipulate different levels or subtypes of guilt (e.g., strong or naturalistic guilt) to change participants' psychological outcomes (e.g., beliefs, attitudes, emotions) and/or behaviors. 3) Non-persuasion intervention with no intention to change participants' beliefs, attitudes, emotions, and/or behaviors.	None was excluded due to this criterion.	(a) Four articles reported no studies that manipulated guilt induction as an independent variable (*n* = 4). (b) Seven articles reported studies that manipulated different levels or subtypes of guilt (*n* = 7).
Comparators	Emotion-neutral control conditions without induction of guilt or any other emotions Exclude: Other emotions or subtypes of guilt.	1,014 articles reported studies that compared the effects of guilt with other emotions, mixed emotions, or different levels or subtypes of guilt without a true control condition (e.g., low guilt) (*n* = 1,014).	None was excluded based on this criterion.
Outcomes	Guilt and any other psychological outcomes, and behavior of participants that are influenced by interventions, including attitude, behavioral intention, behavior, guilt, other emotions, motivation, cognition, efficacy. The outcomes must be directly measured on participants. Exclusion: 1) Guilt as the only outcome. 2) Indirect measurement of outcome.	Four articles reported studies that measured guilt as the only outcome variable (*n* = 4).	One article reported studies that only reported the outcomes based on indirect rating or observation results by non-participant judges (*n* = 1).
Study design	Quantitative experiments using either a pretest-posttest or a posttest-only between-subject control group design in which each participant is assigned to either the intervention or the control group.	92 studies reporting non-quantitative studies only (*n* = 92).	(a) One article reported no studies that used a between-subject design with a guilt appeal and a neutral control condition (*n =* 1).

^a^The first round of screening also excluded five articles not written in English.

^b^The second round of screening also excluded 22 articles that reported no usable statistical results for effect size calculation.

An initial literature search was conducted in 2020 through the following databases: *PsycINFO, ProQuest Dissertations & Theses Global, ERIC, MEDLINE*, and *Communication and Mass Media Complete* via EBSCO. The search terms “Guilt^*^” AND [“appeal” OR “campaign” OR “adverti^*^” OR “message” OR “intervention”] were used. Publicly available doctoral and master's degree theses were also retrieved. A total of 1,240 citations were exported to a citation manager. The initial sample included studies published from 1929 to 2020. To avoid file drawer issues, authors and experts in the field were consulted using the listserve “Communication Research and Theory Network's (CRTNET)” to solicit unpublished studies in their personal libraries, and responses were accepted throughout 2020. This procedure yielded no additional articles. After removing duplicates (*n* = 72), 1,168 articles remained in the initial screening phase.

#### Inclusion and exclusion criteria

**Figure 2:**
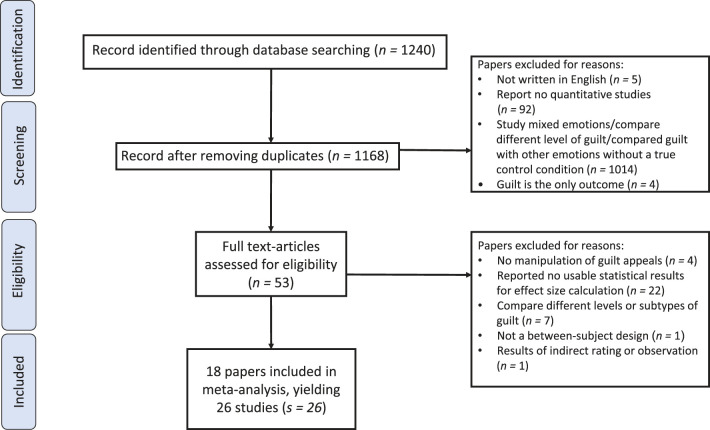
PRISMA flow chart.

Figure 2 provides a PRISMA flowchart indicating the overall inclusion and exclusion procedure. Inclusion and exclusion criteria were developed and verified based on PICOS. The screening results are summarized in Table 1. In the first round, two coders screened the abstracts of 1,168 articles (*n* = 1,168) and excluded (a) five articles (*n* = 5) not written in English (*n* = 5); (b) the comparator criterion: 1,014 articles reported studies that compared the effects of guilt with other emotions, mixed emotions, or variants of guilt without a true control condition (*n* = 1,014); (c) the outcomes criterion: four articles reported studies that measured guilt as the only outcome variable (*n* = 4); and (d) the study design criterion: 92 studies reporting non-quantitative studies only (*n* = 92). A total of 53 articles remained after the first round of screening.

In the second round of screening, the coders further assessed the full text of the remaining articles based on the PICOS criteria and removed the following articles: (1) the population criterion: no articles were excluded (*n* = 0) due to no criterion placed on demographic characteristics; (2) the interventions criterion: (a) four articles reported no studies that manipulated guilt appeals as an independent variable (*n* = 4) and (b) seven articles reported studies that manipulated different levels or subtypes of guilt (e.g., strong or naturalistic guilt) (*n* = 7); (3) the comparators criterion: (a) no articles were excluded due to not reporting at least one guilt appeal condition and one emotion-neutral control condition (*n* = 0); (4) the outcomes criterion: one article with studies that only reported the outcomes based on indirect rating or observation results by non-participant judges (*n* = 1); and (5) the study design criterion: (a) one article reported studies that did not use a between-subject design with a guilt appeal and a neutral control condition (*n* = 1).

In addition, the articles that reported no usable statistical results for effect size calculation were excluded, including (a) 20 articles with studies that provided no statistical results used to calculate effect sizes (i.e., mean and standard deviation, *F*-test values, *t*-test values, Cohen's *d*, and Pearson's *r* values) (*n* = 20), and (b) two articles (*n* = 2) with studies that only reported omnibus *F*-tests for the comparison of multiple conditions (e.g., *F*-tests of guilt, shame, hope, and control conditions) without reporting mean and standard deviation of guilt and neutral control conditions. Studies reported only unstandardized coefficients from multiple regression were excluded because effect sizes computed from these coefficients would be partial and under the influence of other predictors in the multiple regression (Hunter and Schmidt, 1990). Thus, coefficients did not reflect the meaningful effects unless corresponding correlation coefficients were obtained (Peterson and Brown, 2005).

A final sample of 18 articles yielding 26 studies was included in further analyses. The references of all included articles are listed in Appendix C.

#### Coding procedure

Appendix A lists the coded variables and their operational definitions. In the present meta-analysis, we also coded demographic and descriptive information as situational moderators of the included studies (i.e., publication type, sampling frame, funding source, study location, and year of publication). The studies were coded independently by two coders. The coders were graduate students who had completed courses and conducted relevant research projects related to persuasion and emotions. Each coder was responsible for half of the studies. Seven studies (27% of all selected studies) were sampled to compute the intercoder reliability. Intercoder reliability was computed with Krippendorff (2004) alpha (α) for categorical variables and intraclass correlation (*ICC*) for continuous variables (Shrout and Fleiss, 1979). Intercoder reliability of coded variables ranged from 0.74 to 1.00 (α bar = 0.95, *ICC* = 0.87) within the category of agreement (Landis and Koch, 1977). Intercoder reliability was also assessed during the coding of the full sample (Lacy and Riffe, 1996; Neuendorf, 2017). All rating disagreements were resolved through discussion until 100% agreement was achieved for each variable.

#### Calculation of effect size

Appendix B explains how effect sizes were calculated. Because studies of relatively small sample sizes were included, Hedges' *g* was applied to indicate effect size, correcting for small sample sizes (Hedges, 1981; Hedges and Olkin, 1985). A positive Hedges' *g* indicated an increase of the effect on study outcomes was elicited by guilt appeals compared to an equivalent non-guilt or true control condition; and a negative Hedges' *g* indicated a decrease of the effect was elicited by guilt appeals compared to an equivalent non-guilt or true control condition. For studies where the guilt appeal was linked to a reduction in outcome variables for which a greater score indicated negative effects, the computed sign of the effect size was reversed, so all positive differences reflected an increase in the dependent variables.

#### Overall analysis and outliers

Outliers are frequently present in meta-analyses and can distort pooled effect estimates of final effects; therefore, outliers were detected and removed from analyses (Hedges and Olkin, 1985; Schmidt et al., 1993; Viechtbauer and Cheung, 2010). We determined an effect size as an outlier if its confidence interval did not overlap with the confidence interval of the pooled effects. Such an effect size was less likely to be part of the population of pooled effects by the present meta-analysis (Viechtbauer and Cheung, 2010). Using the R package “*altmeta*” (Lin and Chu, 2016), we identified two outliers (i.e., 1.87 and 2.21), which were from Allard and White (2015; Studies 2 and 5).

The removal of these outliers yielded 127 effect sizes from 26 studies that examined the effects of guilt appeals with multiple measurements in the current meta-analysis. For most studies included, we extracted more than one effect size. Considering potential interdependency among effect sizes from the same study, we conducted all moderation analyses using the Robust Variance Estimation (RVE; Gleser and Olkin, 2009; Hedges et al., 2010; Pustejovsky and Tipton, 2022) for adjusting for effect size tendencies while retaining appropriate Type I error rates. All analyses were performed in R (version 4.2.1). We used the “*robumeta*” package (Fisher and Tipton, 2015) to perform the overall and moderator analyses, including meta-regression modeling. All test statistics (i.e., *Q* values) were conducted using the “*metafor*” package (Viechtbauer, 2010). Pairwise comparisons were performed using the “*ClubSandwich*” package (Pustejovsky and Tipton, 2018, 2022; Pustejovsky, 2020) and “*multicomp*” package (Bretz et al., 2010). Appendix D of the Supplemental material lists the acronyms in this article.

## Results

### Description of studies

A total of 127 effect sizes from 26 studies (*N* = 7,512) were included in the meta-analysis. Sample sizes ranged from 61 to 1,054 (*M* = 288.90, *SD* = 261.22). Participants' mean age ranged from 19.34 to 54 years (*M* = 28.68, *SD* = 11.65).

### Publication bias

**Figure 3:**
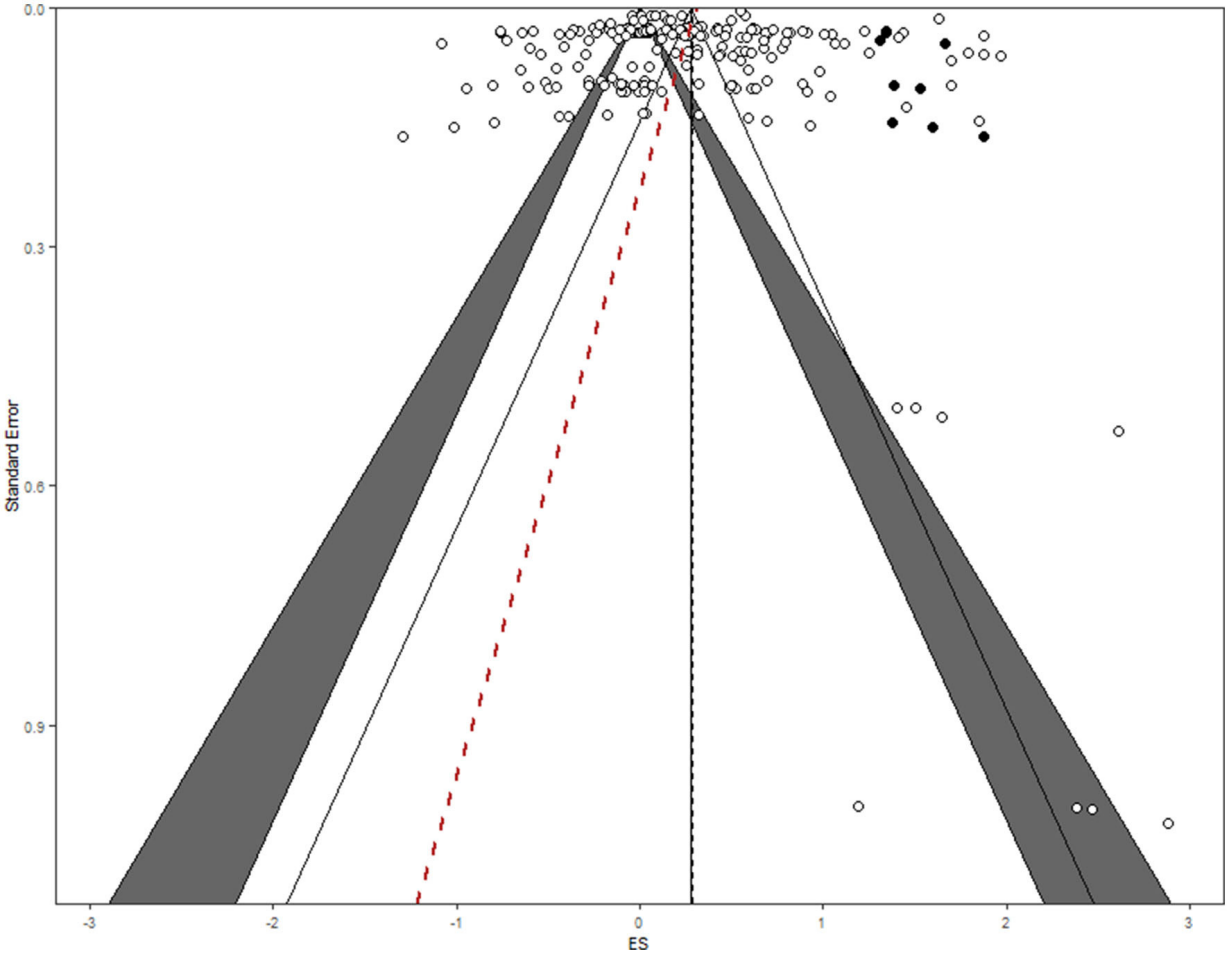
Funnel plot with trim and fill and Egger's regression.

The rank correlation test (Kendall's *tau*) of Begg and Mazumdar (1994) suggested a non-significant relationship between standard error and effect size (Kendall's *tau* = 0.08, *p* = 0.17), indicating no publication biases. In addition, Egger's regression (Sutton, 2009) test showed a non-significant relationship between standard error and effect size (*z* = 0.24, *p* = 0.81), which indicated no evidence for publication bias. The trim and fill method of Duval and Tweedie (2000) suggested that zero studies were missing on the right side of the funnel plot (*SE* = 6.43). The graphical evaluation of the funnel plot (Figure 3) shows a symmetrical inverted shape, indicating that the effects of the included studies were variable and scattered to both sides of the point estimate of effect. Thus, the appearance of the funnel plot suggests no missing studies, either due to publication bias or sampling errors. Rosenthal's (1979) fail-safe *N* was 6,035 at the observed significance level of 0.0001 and the perceived significance level of 0.05. We thus concluded that it is unlikely that this meta-analysis missed a substantial number of studies. Given these results, we concluded that potential publication biases were absent in extant literature examining the effects of guilt appeals. A funnel plot with trim and fill and Egger's regression is provided in Figure 3.

### Overall analysis

Excluding outliers, the overall effect size of guilt appeal produced on all types of outcomes was significant, *Q*(126) = 902.31, *t*(24.2) = 3.11, *I*-squared = 91%, *tau*-square = 0.24 (*SE* = 0.04), with *g* = 0.19, *SE* = 0.05, *rho* = 0.8, 95% CI (0.10, 0.28), *p* < 0.001. The results indicate the heterogeneity in true effect sizes of guilt appeal (Higgins et al., 2003). Thus, we used the random-effects model in the analysis. Providing supportive evidence to address RQ1, this finding suggested a small effect size of guilt appeals. In contrast, the overall effect size of guilt appeal, including outliers, was 0.24, *p* < 0.001, suggesting that having outliers in subsequent analyses would inflate the overall effect size. The *post-hoc* statistical power of the overall analysis was 80%. Thus, without additional studies, the sample size was large enough to detect the true effect sufficiently.

### Analysis of moderators

All moderator analyses were conducted for studies with the removal of outlier estimations. We reported the results of individual moderators. In addition, a meta-regression model with theoretical moderators as the predictors was also specified to explain the effects of guilt appeals (see details in Tables 1–**3**).

### Analysis of theoretical moderators

#### Responsibility attribution

Responsibility attribution moderated the effects of guilt appeals. H1a was supported. Guilt appeals that attributed responsibilities to the perceiver produced smaller effects compared to those not attributing responsibilities (Table 2).

**Table 2:** Effect sizes by theoretical moderators (*s*^a^ = 26, *k*^b^ = 127).

**Moderator**	**Unadjusted ḡ**	***s***	***k***	***b***	***SE***	***t***	***Df* ^c^**	**95% CI**	
								**LL**	**UL**
Intercept	–	–	–	0.41	0.29	1.41	1.92	−0.89	1.71
Responsibility attribution	***Q**_***within***_* = 865.80^***^; ***Q**_***between***_* = 24.85^***^								
No responsibility attributed in guilt appeals (description of harm/suffering only) (ref.)	0.36	14	48	–	–	–	–	–	–
Responsibility attributed in guilt appeals	0.08	12	79	−0.28^†^	0.14	−1.97	9.74	−0.60	0.04
Controllability attribution	***Q**_***within***_* = 899.43^***^; ***Q**_***between***_* = 16.32^***^								
No controllable causes attributed in guilt appeals (ref.)	0.22	8	22	–	–	–	–	–	–
Controllable causes attributed in guilt appeals	0.18	18	105	−0.15^*^	0.39	−0.38	1.47	−2.54	2.24
Stability attribution	***Q**_***within***_* = 898.61^***^; ***Q**_***between***_* = 17.40^**^								
No unstable causes attributed in guilt appeals (ref.)	0.09	10	36	–	–	–	–	–	–
Unstable causes attributed in guilt appeals	0.23	16	91	0.18	0.13	1.38	5.75	−0.14	0.49
The proximity of perceiver-victim relationship	***Q**_***within***_* = 896.44^***^; ***Q**_***between***_* = 17.12^**^								
Self/personal relationship (ref.)	0.20	8	26	–	–	–	–	–	–
Work/professional relationship	−0.03	1	2	0.42	0.48	0.87	3.38	−1.03	1.86
Strangers	0.15	9	65	−0.12	0.37	−0.33	1.61	−2.13	1.89
Non-human/other	0.27	8	34	0.13	0.39	0.32	2.13	−1.45	1.70
Provided recommendation of reparative behaviors	***Q**_***within***_* = 834.98^***^; ***Q**_***between***_* = 22.78^***^								
No (ref.)	−0.20	3	8	–	–	–	–	–	–
Yes	0.22	23	119	0.56	0.26	2.19	2.23	−0.44	1.56
Outcome types^d^	***Q**_***within***_* = 836.19^***^; ***Q**_***between***_* = 27.57^**^								
Guilt (ref.)	0.46	–	18	–	–	–	–	–	–
Attitude	0.14	–	33	−0.77^**^	0.20	−3.89	13.52	−1.20	−0.34
Behavioral intention	0.29	–	31	−0.68^**^	0.17	−3.99	13.12	−1.05	−0.31
Behavior	0.16	–	14	−0.58^*^	0.25	−2.37	9.44	−1.13	−0.03
Emotion other than guilt	−0.05	–	9	−0.72^†^	0.30	−2.38	6.66	−1.42	0.002
Motivation	0.33	–	2	−0.64^†^	0.17	−3.77	1.80	−1.45	0.17
Cognition	−0.01	–	20	−0.65^**^	0.17	−3.92	10.95	−1.01	−0.28

^***^p < 0.001; ^**^p < 0.01; ^*^p < 0.05; ^†^*p* < 0.10.

^a^Indicates number of studies.

^b^Indicates number of effect sizes.

^c^Do not trust t value if *df* < 4.

^d^Only displays numbers of effect sizes.

#### Controllability attribution

The meta-analysis supported H1b, indicating that controllability attribution moderated persuasive effects on outcomes. Guilt appeals depicting controllable causes generated smaller effects than those not depicting controllable causes.

#### Stability attribution

Stability attribution moderated the effects of guilt appeals, supporting H1c. Guilt appeals attributing the victim's suffering to an unstable cause produced greater effects on outcomes than did those not depicting such causes.

#### The proximity of perceiver–victim relationship

The proximity of the perceiver–victim relationship moderated the persuasive effects of guilt appeals, supporting H2. Guilt appeals featuring the victim in a personal relationship with the perceiver or being a non-human subject generated greater persuasive effects than those featuring victims in other relationships.

#### Recommendation of reparative behaviors

The results supported H3. When recommending reparative behaviors, guilt appeals produced significantly stronger effects than those without recommending reparative behaviors.

#### Outcome types

H4 was supported, indicating varied effects of guilt appeals by different types of outcomes. We found that guilt appeals were more effective in eliciting guilt, attitude, behavior, behavioral intention, and motivation.

#### Meta-regression of theoretical moderators

We conducted meta-regression for theoretical moderators. All theoretical moderators were included in the same meta-regression model because the influence of these moderators could occur simultaneously (Roseman, 2013). After adjustment for other theoretical moderators, the effects of guilt appeals were significantly moderated by controllability attribution and measured outcomes but marginally lowered by responsibility. Other theoretical moderators were not significant. After adjustment for other theoretical moderators, guilt appeals that attributed the suffering to the responsibility of a perceiver and to controllable causes produced smaller effects than those without these attributional factors. The effect size of guilt appeals depended on outcome types as stronger effects were mostly produced on behavioral intention and behavior.

### Analysis of methodological moderators

The meta-analysis evaluated the following methodological moderators of guilt appeals to answer RQ2 (see Table 3).

**Table 3:** Effect sizes by methodological moderators (*s*^a^ = 26, *k*^b^ = 127).

**Moderator**	**Unadjusted ḡ**	***s***	***k***	***b***	***SE***	***t***	***Df* ^c^**	**95% CI**	
								**LL**	**UL**
Intercept	–	–	–	−0.41	0.29	−1.39	6.65	−1.11	0.29
Contexts	***Q**_***within***_* = 837.83^***^; ***Q**_***between***_* = 25.26^***^								
Advertising/marketing (ref.)	−0.11	2	14	–	–	–	–	–	–
Education	0.34	7	31	0.65^*^	0.27	2.41	6.35	−0.001	1.31
Environment-related	0.23	11	62	0.51^†^	0.21	2.48	3.79	−0.07	1.10
Medical/health-related	−0.01	2	4	0.35	0.36	0.98	3.56	−0.69	1.39
Other	0.06	4	16	0.22	0.21	1.07	3.29	−0.41	0.86
Induction methods	***Q**_***within***_* = 890.40^***^; ***Q**_***between***_* = 21.53^***^								
Persuasive messages (ref.)	0.19	15	90	–	–	–	–	–	–
Imagination and recall	0.35	10	25	0.19	0.30	0.64	5.51	−0.56	0.94
Other	−0.13	1	12	0.28	0.29	0.95	6.65	−0.42	0.98
Narrative	***Q**_***within***_* = 897.51^***^; ***Q**_***between***_* = 18.03^***^								
Non-narrative (ref.)	0.15	15	96	–	–	–	–	–	–
Narrative	0.32	11	31	−0.07	0.14	−0.53	2.14	−0.63	0.48
Modalities	***Q**_***within***_* = 896.04^***^; ***Q**_***between***_* = 18.36^***^								
Unspecified (ref.)	0.08	7	30	–	–	–	–	–	–
Text only	0.32	8	29	0.38	0.29	1.28	6.65	−0.33	1.08
Text and image	0.18	11	68	−0.01	0.34	−0.03	6.80	−0.81	0.79
Time points of measurement	***Q**_***within***_ **=*** 895.31^***^; ***Q**_***between***_ =* 16.96^***^								
Immediate (ref.)	0.21	23	107	–	–	–	–	–	–
Within a week	0.13	2	18	−0.07	0.07	−0.99	1.20	−0.70	0.56
After a week	−0.03	1	2	−0.24^**^	0.07	−3.50	12.55	−0.38	−0.10

^***^p < 0.001; ^**^p < 0.01; ^*^p < 0.05; ^†^*p* < 0.10.

^a^Indicates number of studies.

^b^Indicates number of effect sizes.

^c^Do not trust *t* value if *df* < 4.

#### Contexts of persuasion objects

Effects of guilt appeals varied by contexts of persuasion objects. Guilt appeals were more effective in education and environment-related contexts compared to advertising or marketing contexts. On the other hand, pairwise comparisons showed that guilt appeals did not differ between advertising or marketing and health contexts.

#### Induction methods

Effects of guilt appeals varied by induction methods. Guilt appeals using imagination or recall through interpersonal communication instigated greater effects than persuasive guilt messages.

#### Narrative

The use of narrative or not influenced the effects of guilt appeals. Guilt appeals using narrative elicited more effects than those using non-narrative guilt appeals.

#### Message modalities

Message modalities moderated effects of guilt appeals. Guilt appeals using text elicited stronger effects than text and image-based guilt appeals.

#### Time point of measurement

Different time points of measurement moderated the effects of guilt appeals. The effects of guilt appeals were stronger if measured immediately after the exposure than those measured later within a week or after a week of exposure.

### Analysis of situational moderators

The meta-analysis examined five situational moderators related to the characteristics of published studies. The result showed that published studies with university panels after 2011 were associated with more effects (all *p* < 0.10; Table 4).

**Table 4:** Effect sizes by situational moderators (*s*^a^ = 26, *k*^b^ = 127).

**Moderator**	**Unadjusted ḡ**	***s***	***k***	***b***	***SE***	***t***	***Df* ^c^**	**95% CI**	
								**LL**	**UL**
Intercept	–	26	127	−0.72^†^	0.21	−3.54	2.36	−1.49	0.04
Publication type	***Q**_***within***_ **=*** 854.88^***^; ***Q**_***between***_ =* 19.44^***^								
Unpublished (e.g., master's or doctoral theses) (ref.)	0.01	4	16	–	–	–	–	–	–
Published (e.g., journal or book)	0.22	22	111	0.15	0.17	0.91	3.89	−0.32	0.62
Sampling frame	***Q**_***within***_ **=*** 899.40^***^; ***Q**_***between***_ =* 16.13^**^								
Online panel (ref.)	0.19	8	48	–	–	–	–	–	–
University panel	0.19	16	64	0.30^*^	0.13	2.37	12.47	0.03	0.57
Community sample	0.20	2	15	−0.004	0.17	−0.02	2.26	−0.65	0.64
Funding source	***Q**_***within***_ **=*** 888.02^***^; ***Q**_***between***_ =* 18.18^***^								
No funding (ref.)	0.17	25	114	–	–	–	–	–	–
Funded	0.37	1	13	0.17	17	1.03	4.39	−0.27	0.61
Study location	***Q**_***within***_ **=*** 890.07^***^; ***Q**_***between***_ =* 20.03^***^								
U.S. (ref.)	0.12	17	82	–	–	–	–	–	–
Non-U.S.	0.32	9	45	0.10	0.15	0.62	8.83	−0.25	0.45
Year of report	***Q**_***within***_ **=*** 813.00^***^; ***Q**_***between***_ =* 30.37^***^								
1991–2000 (ref.)	−0.23	2	16	–	–	–	–	–	–
2001–2010	0.08	4	12	0.34	0.21	1.61	1.88	−0.62	1.29
2011- to date	0.27	20	99	0.68^†^	0.18	3.83^**^	1.76	−0.19	1.56

^***^p < 0.001; ^**^p < 0.01; ^*^p < 0.05; ^†^*p* < 0.10.

^a^Indicates number of studies.

^b^Indicates number of effect sizes.

^c^Do not trust t value if df < 4.

## Discussion

The present meta-analysis aimed to conduct a comprehensive synthesis of guilt appeals. Compared with previous meta-analyses (O'Keefe, 2000; Boster et al., 2016; Xu and Guo, 2018; Turner and Rains, 2021), the present study centered on the emotional mechanism of guilt appeals and accounted for multiple theoretical moderators associated with guilt elicitation and coping. Overall, our findings showed a small effect of guilt appeals. The effect of guilt appeals could be influenced by guilt induction and coping processes, which echoed empirical evidence that guilt appeals may not be effective without considering multiple elicitation conditions (e.g., Chang, 2012) or approaches to regulating emotions (e.g., Graton et al., 2016). In addition, we identified various methodological approaches in past research that could moderate the effectiveness. The findings brought forth theoretical and practical implications of designing, testing, and implementing guilt appeals.

### Theoretical moderators of guilt appeals

#### Responsibility attribution

The results showed that guilt appeals without attributing the responsibility to the perceiver produced greater effects compared to those with responsibility. This finding appeared contrary to past research which identified an essential role of responsibility in guilt arousal (Tangney et al., 2007; Roberts et al., 2014; Miceli and Castelfranchi, 2018). We considered two possible explanations. First, without blaming one's behavior as the direct culprit of a problem, guilt appeals could be effective by focusing on the perceiver's social obligations in important social issues and their contribution to potential solutions (Urbonavicius et al., 2019). Some cases of this persuasive approach direct the perceiver to examine their advantages over others and take on social obligations (Chang, 2011; Harth et al., 2013; Dull et al., 2021). Such persuasive effects could be possibly related to existential guilt (Zimmermann et al., 2011; Binder, 2022). Negative feelings might result from a motivational mechanism of guilt intended to promote empathetic concern and altruistic helping behaviors toward other's suffering linked to problems in a social system (Baumeister et al., 1994; Roberts et al., 2014). In this persuasion process, the sense of responsibility was not imposed on the perceiver by the persuader but potentially developed by the perceiver once guilt was aroused (Berndsen and Manstead, 2007).

Second, attributed causes by guilt appeals might not be consistent with how responsibility was appraised by the perceiver, which could hinder the persuasive effects. The theoretical review shows that responsibility ambiguously involves appraisals of causality, intentionality, and capacity (Malle et al., 2014). One might accept themselves as the causal agent of a negative event but could still avoid the blame by justifying their actions or denying the nature of such events (Malle et al., 2014; Gerstenberg et al., 2018). Thus, attributed responsibilities in guilt appeals might oversimplify how people evaluate the cause of a specific event and whether they accept the blame (Malle et al., 2014).

Because responsibility is difficult to pinpoint in many situations (Tangney et al., 2007), assigning responsibility in guilt appeals should warrant careful consideration. While guilt appeals could emphasize perceived responsibility based on the moral values in a social system, personal traits and internalization of such values can also change how much responsibility one would accept (Ho et al., 2004; Tangney et al., 2007; Malle et al., 2014). Thus, the main takeaway is that the suffering of the victim should be the focus of guilt appeals as explicit attribution of responsibility is not necessarily accepted by the perceiver. Future research can also emphasize potential connections between the perceiver and the victims to stimulate empathy and promote a sense of equity (Harth et al., 2013). It may also be beneficial to test whether the evaluative value underlying a guilt-arousing event is consistent with the moral system held by the target population, especially when the contexts of guilt appeals are familiar to the perceiver (Jennings et al., 2015).

#### Stability and controllability attributions

Guilt appeals that attribute the causal event to unstable factors were more effective than those without such attributional information. This finding confirmed unstable factors as a key distinguishing dimension between guilt and other moral-related emotions, such as shame (Tangney et al., 2007; Greenbaum et al., 2020). Guilt appeals are designed to modify the perceiver's changeable behaviors or beliefs, rather than more stable characteristics, such as personality (Xu and Guo, 2018). Without specifically attributing the causes to be changeable, guilt appeals could not sufficiently stimulate the perceiver's adaptive response, such as reparation and change (Tangney et al., 2007; Shen, 2018; Graton and Mailliez, 2019).

Nonetheless, guilt appeals emphasizing the causes to be controllable by the perceiver might decrease persuasive effects. This finding could be explained by the relatedness of different attributional dimensions in guilt appeals. Theoretical reviews indicate that the perceiver's behavior, an internal, unstable aspect of the self, is a necessary condition of guilt induction (Tracy and Robins, 2006; Tangney et al., 2007; Roberts et al., 2014). Guilt appeals also seek reparative behaviors to undo transgressions (Antonetti et al., 2018; Graton and Mailliez, 2019). Thus, the controllability of the cause may have been suggested by the induction- and coping-related information in a guilt appeal, including attributions of responsibility and stability as well as reparative behaviors (Tracy and Robins, 2006). For example, a guilt appeal directs the perceiver to appraise their consumer behaviors of traditional printers as the cause of environmental pollution and advocates them to use environment-friendly printers as the reparation (Chang, 2012). This case of guilt appeals has suggested that the cause of a negative event can be controlled by the action taken by the perceiver. As such, arguments reinforcing controllability may be redundant and further raise psychological reactance among the perceivers (Graton et al., 2016). On the other hand, controllability might not be appraised until the perceiver has evaluated and accepted other attributions in a guilt appeal (Malle et al., 2014). Given these potential explanations, future research is warranted to confirm the effects of controllability in guilt appeals.

#### The proximity of perceiver–victim relationship

Contrary to previous literature (Ghorbani et al., 2013), our analysis did not suggest that guilt appeals would be necessarily more effective by portraying a victim in a closer, more personal relationship with the perceiver. One explanation is that personal relationships do not necessarily reflect the psychological distance between the perceiver and another person or object (Trope and Liberman, 2010). In addition, the perceiver might maintain a broader sense of duty and personal meaning in a human society that exists beyond common personal relationships (Binder, 2022). The perceived relatedness on a more expansive level could evoke guilty feelings when personally unrelated individuals or the natural environment are impacted by a negative event, motivating helping and prosocial behaviors (Zimmermann et al., 2011). Thus, additional research is needed to evaluate and incorporate perceived closeness to the victim or an object by the perceiver in guilt appeals. Concrete information about the victims may also improve the perceived proximity (Ghorbani et al., 2013).

#### Recommendation of reparative behaviors

Recommending reparative behaviors increased the effects of guilt appeals. This result supported the importance of reparative actions as a coping process in which guilty people control negative feelings by making amends (Ketelaar and Tung Au, 2003; Tangney et al., 2007; Shen, 2018; Vaish, 2018). Our interpretation is that guilty perceivers were more likely to seek readily available information in the stimuli, rather than from alternative sources, that can help them quickly assuage the feeling (Tangney et al., 2007; Graton and Mailliez, 2019). Recommending reparative behaviors might also emphasize the repairable nature of the cause (Tangney et al., 2007), which increases the efficacy of making restitution. In contrast, non-repairable outcomes can cause maladaptive responses, lowering persuasive effectiveness (Kubany and Manke, 1995; LeBlanc et al., 2020). Based on current findings, practitioners using guilt appeals should provide a solution to rectify negative situations, especially when a behavior is not commonly considered wrong.

#### Outcome type

Expanding previous meta-analyses, our study tested the effectiveness of guilt appeals on various types of outcome variables. We found significant effects of guilt appeals on guilt, attitudes, and behavioral outcomes in support of broad influences of guilt as a persuasive approach. The strong effects on guilt reflect the theoretical foundation of guilt appeals, which centers on guilt as an emotional mechanism of persuasion (O'Keefe, 2002; Antonetti et al., 2018; Graton and Mailliez, 2019). Attitudinal and behavioral outcomes changed by guilt appeals showed that the influence on how one thinks and behaves could function as a key mechanism to regulate guilt and elicit persuasive outcomes (Tangney et al., 2007; Scherer and Moors, 2019).

#### Meta-regression of theoretical moderators

Overall, the meta-regression finding suggests that most of the factors could not consistently influence the effects of guilt appeals. After controlling for other theoretical moderators, stability attribution, proximity of perceiver–victim relationship, and recommendation of reparative behaviors did not significantly moderate the effects of guilt appeals. It is possible that the persuasive effects of guilt appeals were potentially generated through the appraisals of various factors in a correlated and sequential manner (Tracy and Robins, 2006; Tangney et al., 2007; Malle et al., 2014). Thus, one of these factors could be a necessary but insufficient condition of guilt arousal and persuasive outcomes. However, after adjustment for other theoretical covariates, guilt appeals emphasizing responsibility and controllability lowered persuasive effects, which further confirmed our previous finding. The perceiver might feel guilty about the other's suffering and engage in reparative behaviors based on holistic descriptions of the event but explicit information about the responsibility and controllability of the cause could backfire.

Moreover, insignificant theoretical moderators in the meta-regression also suggested that cognitive appraisals are subject to individual differences. The cognitive system that activates guilt depends on situational and individual differences, which increases uncertainty in the appraisal and regulation process (Muir et al., 2017; Kobylińska and Kusev, 2019). Future research should address whether personal preferences and certain persuasion contexts can influence how different attributions are weighed and prioritized in the appraisal of guilt appeals (Mantler et al., 2003). The finding also challenges the universal standard of guilt appeals that would fit all situations and individuals. How to design guilt appeals that involve different attribution dimensions for various contexts is an important question to answer.

### Methodological moderators

#### Contexts

This study is the first meta-analysis to compile published evidence across several different contexts where guilt appeals are commonly tested. Guilt appeals were most effective when employed in environmental advocacy and education. This finding further demonstrates that guilty feelings are linked to one's moral and social responsibility, which motivate caring and prosocial behaviors for the wellbeing of others and the entire society (Zimmermann et al., 2011). However, guilt appeals used in health-related contexts were less effective than the control condition, which conflicts with Xu and Guo's (2018) meta-analysis. The discrepancy was possibly due to the fact that our analysis examined more outcomes influenced by guilt appeals in health-related contexts (e.g., efficacy and perceived threat) than only attitude and behavioral intentions assessed by Xu and Guo's study. In addition, this meta-analysis investigated the persuasive effects of guilt appeals on health behaviors benefiting both oneself and others (e.g., blood donation).

#### Induction methods

We found that compared with persuasive messages, imagination and recall as an induction method increased the effects of guilt appeals. The finding was consistent with a theoretical argument that emotion enhances experiential memory (Gomes et al., 2013). People can better remember an emotionally valenced event relative to neutral ones, which is encoded in memory to reconstruct an episode of past experience (Finn and Roediger, 2011). Thus, guilt-arousing events can be effectively consolidated in memory and easily retrieved once recalled, which may generate a consistent influence on metacognitive judgment. Through recall, people would examine the role of themselves in a negative event. This process could help trigger self-conscious emotions such as guilt (Tracy and Robins, 2006). This finding further supported earlier calls for using recall or imagination to elicit guilt and reach the persuasive goal in interpersonal settings (Baumeister et al., 1995; O'Keefe, 2002). Future studies should replicate the effectiveness of inducing guilt through recall in real-world settings. On the other hand, message designers should find the connection and relevance of the present object to the perceiver's emotional experience. Practitioners may also elicit guilt by encouraging the perceiver to explore personal experiences and examine their own transgression.

#### Narrative

Guilt appeals involving narrative were found relatively more effective. This result suggested the advantage of narrative in elevating emotional experiences (Nabi and Green, 2015). The practice of storytelling could engage the perceiver to reflect upon their own experience and increase understanding of the victim's suffering (Hamby et al., 2017). In addition, narrative can help the perceiver identify with the victim and find similarities between themselves and others (Hoeken et al., 2016). These processes could heighten one's self-representation in a social or moral context, which facilitates guilt arousal (Tracy and Robins, 2006).

Because guilt appeals are sometimes perceived to be manipulative (Cotte et al., 2005; Graton et al., 2016), narratives can also implicitly express persuasive purposes without increasing reactance (Reynolds-Tylus, 2019; Hasell et al., 2020). However, narrative persuasion might be less effective if persuasive arguments are incongruent with the perceiver's existing values because narrative engagement and enjoyment could be interrupted by counterarguments (Slater and Rouner, 1996; Krakow et al., 2018). Thus, non-narrative guilt appeals for mainstream social or moral causes might still be advantageous. Future research and practice can test the effects and limitations of narrative vs. non-narrative guilt appeals, especially when promoting controversial or less accepted topics.

#### Modalities

Text-based guilt appeals generated the strongest effects, which could be due to in-depth cognitive processing enabled by textual information (Mayer, 2014). As a result, the perceiver receiving textual information could inspect the claims of guilt appeals closely before accepting the request for compliance. This finding lent support to the continuing use of textual information as the major approach to developing guilt appeals. Using both text and image in guilt appeals produced lower but consistent effects. While effective images should attract attention and provide resemblances to real-life experiences, some images used in guilt appeals may not be congruent with textual information, hampering effective understanding (van Rompay et al., 2010; Sung and Mayer, 2012). Researchers should also assess whether new modalities facilitate the cognitive or emotional processing of guilt appeals that can elevate attention or guilt arousal (Geise and Baden, 2015; Kandaurova and Lee, 2019).

#### Time points of measurement

The meta-analysis showed the effects of guilt appeals waned after time passed. Despite recent literature (Antonetti et al., 2018; Kemp, 2023), the delayed effects of guilt appeals were found to be lower across all studies. Considering the transient nature of guilty feelings after misdeed (Brosch, 2021; Ogunfowora et al., 2023), the effects of guilt appeals should be sought immediately after persuasion. Nonetheless, we could not rule out the possibility that delay effects still exist (Brosch, 2021) but could not be observed by existing measurements. Thus, future research should explore alternative mechanisms and measurement approaches for enduring persuasive effects.

### Limitations and future research

This meta-analysis has three limitations. First, the sampling part of the meta-analysis was conducted in 2020, and no studies published since then were sampled. In addition, meta-analyses need to maintain a balance between the study design and the scope of sampling because the validity of the study findings depends on whether we synthesized comparative studies in the literature (Cortina, 2003). Thus, we carefully developed the inclusion/exclusion criteria based on the research purpose and research questions and strictly enforced these criteria in the coding process. As a result, many studies, including recent ones, that failed to meet the inclusion and exclusion criteria were excluded from the final sample. For instance, studies that used mixed emotions in persuasive appeals were excluded because the effects of guilt appeals would not be comparable to their combination with another emotion due to distinct appraisal antecedents. As shown in the results, the present meta-analysis had reached sufficient power to examine the true effects of guilt appeals. The findings based on more rigorous conceptual and operational processes could help address the gap in the previous meta-analyses. Nonetheless, future research should sample more recent published and unpublished studies to replicate the findings of this meta-analysis. Second, because guilt is rooted in one's connection with other people and social relationships (Tangney et al., 2007), people may feel guilty about different issues and adopt new coping approaches as society and cultures evolve. These changes introduce new ways to design guilt appeals and test their effects. Thus, future studies can examine additional moderators involved in guilt induction, coping, and methodological approaches to advance this area of research. Third, through a synthesis of past studies, this meta-analysis provided important insights into the true effects of guilt appeals and their moderators. Compared with individual studies, meta-analyses make a unique contribution to the literature by examining and synthesizing a complex, even contradictory body of literature in an effect (Sutton and Higgins, 2008; Haidich, 2010). Nonetheless, researchers can also conduct empirical studies to confirm whether the significant moderators in this study could improve the effects of guilt appeals.

## Conclusion

The present meta-analysis was a comprehensive evaluation of the effectiveness of guilt appeals. The findings of the present meta-analysis showed an overall small effect of guilt appeals, and this effect could be stronger on guilt emotions, attitudes, behaviors, and behavioral intentions. Guilt appeals were particularly more effective in the contexts of environmental advocacy and education. The study contributes to the understanding of the specific theoretical (i.e., responsibility attribution, controllability attribution, stability attribution, and recommendation of reparative behaviors and outcome types) and methodological factors (i.e., contexts, induction methods, narrative, message modalities, and time point of measurement) that could facilitate or impede the persuasive effects.

From a practical perspective, the findings could improve the confidence of practitioners in using and developing guilt appeals by offering valuable insights into different parts and processes of this persuasive means. To achieve better effectiveness, practitioners might design guilt appeals that portray the victim's suffering, focus on the unstable aspects of the cause, and offer reparative recommendations. Conversely, it is advisable to avoid arguments that attribute the victim's suffering to the perceiver's responsibility or overly emphasize the controllability of the cause as these approaches might be perceived as manipulative and adversely affect persuasion outcomes. Moreover, specific methods should be considered in practice to improve the effectiveness of guilt appeals, including invoking imagination and recall to elicit guilt, employing narratives, and incorporating textual information. Immediate persuasive effects should also be sought in practice.

## Data availability statement

The original contributions presented in the study are included in the article/Supplementary material, further inquiries can be directed to the corresponding authors.

## Author contributions

WP: conceptualization, methodology, writing—original draft, and writing—review and editing. QH: methodology, formal analysis, software, writing—original draft, and visualization. BM and DL: methodology and investigation. EM and JS: conceptualization and writing—original draft. NC: conceptualization, writing—review and editing, and supervision. All authors contributed to the article and approved the submitted version.
