# Supplementary material for: When guilt works: a comprehensive meta-analysis of guilt appeals
Source: Front Psychol. 2023 Sep 28;14:1201631. doi: 10.3389/fpsyg.2023.1201631 (PMC10568480; doi:10.3389/fpsyg.2023.1201631)
Supplement: Supplementary file 1 [file Presentation_1.pdf]

Supplemental File

Appendix A: Coded Variables, Operational Definitions, and Coding Instructions

For each included article, the following variables are recorded by two independent coders when information is available.

Specifically, variables of study characteristics include:

A. **Publication type** (1= Published (e.g., journal, book chapter) 2 = Unpublished manuscript (e.g., master's and doctoral theses))

   Operational definition and coding instructions:

   This variable records the type of publication of the sampled research article.

   (1) Published: The research article has been published in an academic journal, book, or other publication outlets.

   (2) Unpublished: The research article has not been published in a journal, book, or other publication outlets. Master's or doctoral theses are considered as unpublished.

B. **Sampling frame** (1 = Online panel; 2 = University panel; 3 = Community; *4 = Other; and *5 = Unknown);

   Operational definition and coding instructions:

   This variable is to record the source of sampling study participants. The information provided by each study is coded.

   (1) Online panel: The study samples participants from online sources. The sampling and recruitment process is completed online.

   (2) University panel: The study samples participants from university campuses. Participants are college students.

(3) Community: The study samples participants from local communities outside university campuses.

(4) Other: The study samples participants from a source other than online, university, and community.

(5) Unknown: The study does not provide the details of sampling frame.

C. **Funding source** (1 = No funding; 2 = Federal/National Government; *3 = State/Local agency; *4 = Foundation; and 5 = Other). Note: Categories have been combined into “No funding” and “Funded.”

Operational definition and coding instructions:

This variable records the source from which authors of the research article receive funding support.

(1) No funding: The authors of the research article received no funding support. If the authors did not indicate whether they receive funding support or not, this option is coded.

(2) Federal/National Government: The authors of the research article received funding support from the Federal Government in U.S. or the national government of another country.

(3) State/Local Government: The authors of the research article received funding support from a state or local government in the U.S. or from equivalents in another country.

(4) Foundation: The authors of the research article received funding support from non-governmental organizations or foundations.

(5) Other: The authors of the research article received funding support from a source

that does not belong to the categories above.

D. **Study location** (1 = US; and 2 = Non-U.S.; and *3 = Unknown).

Operational definition and coding instructions:

This variable records the location where the study was conducted. If the sampling location is different from that of the affiliated university of the authors, code the sampling location. If no location is reported in the article, the location of the affiliated university of the first author is coded.

E. Year of report

Operational definition and coding instructions:

This variable records the year when the study was published.

F. Sample size

Operational definition and coding instructions of variable E, F, G, and H:

The coders record the information provided by each study that describes the percentages of women, non-White participants in the sample, mean age of the sample, and the sample size.

Guilt appeal variables include:

A. **Responsibility Attribution** (1 = Responsibility attributed in a guilt appeal; 2 = No responsibility attributed in a guilt appeal/description of harm or suffering only):

Operational definition and coding instructions:

The variable records whether the guilt appeal in a study attributes the suffering of others to the perceiver’s action or belief.

(1) Responsibility attributed in a guilt appeal: The guilt appeal in a study attributes the suffering of the victims to the perceiver’s action or belief.

(2) No responsibility attributed in a guilt appeal/Description of harm or suffering only: The guilt appeal in a study describes the harm on victims or their suffering but does not attribute the harm to the perceiver's actions or beliefs. If the study does not explicitly indicate the responsibility attribution, this option is selected.

B. **Controllability Attribution** (1 = Controllable causes attributed in guilt appeals; 2 = No controllable causes attributed in guilt appeals):

Operational definition and coding instructions:

This variable records whether the guilt appeal in a study attributes the victim's suffering to a cause that the perceiver has control over.

Controllable causes attributed in guilt appeals: The guilt appeal in a study attributes the victim's suffering to a cause that the perceiver has control over.

No controllable causes attributed in guilt appeals: The guilt appeal in a study does not attribute the victim's suffering to a cause that the perceiver has control over. If the study does not explicitly indicate the controllability attribution, this option is selected.

C. **Stability Attribution** (1= Unstable causes attributed in guilt appeals; 2 = No unstable causes attributed in guilt appeals):

Operational definition and coding instructions:

This variable is to record whether the guilt appeal in a study attributes the victim's suffering to a cause that is temporary and changing.

(1) Unstable causes attributed in guilt appeals: The guilt appeal in a study attributes the victim's suffering to a cause that is temporary and changing.

(2) No unstable causes attributed in guilt appeals: The guilt appeal in a study does not attribute the victim's suffering to a cause that is temporary and changing. If the

study does not explicitly indicate the stability attribution, this option is selected.

D. **The Proximity of Perceiver-Victim Relationship** (1 = Self/Personal relationship; 2 = Work/Professional relationship; *3 = Community/Acquaintance; 4 = Strangers; and 5 = Other/ Non-human):

Instructions for coder:

This variable records the social distance between the perceiver and the victim as described in the study.

(1) Self/Personal relationship: The study describes the victim to be self or someone close to the perceiver, such as a family member or friend.

(2) Work/Professional relationship: The study describes the victim to be a workplace co-worker, supervisor, subordinate, or in any other professional relationship with the perceiver.

(3) Community/Acquaintance: The study describes the victim to be someone that the perceiver knows slightly but does not have a professional or personal relationship with. The victims may also be someone that lives in the same neighborhood with the perceiver.

(4) Strangers: The study describes the victim to be someone that the perceivers have not met or known before. The victim has no direct connection or relationship with the perceiver.

(5) Other/Non-human: The study describes the victim to be a non-human entity, such as the natural environment.

E. **Recommendation of reparative behaviors** (1 = Yes; 2 = No):

Operational definition and coding instructions:

The variable is to record whether the guilt appeal in a study recommends any counteracting behavior or measures intended to undo the perceiver's wrongdoing and reduce its harm on victims.

(1) Yes: The guilt appeal in a study recommends at least one counteracting behavior or measure to undo the perceiver’s wrongdoing and reduce its harm on victims.

(2) No: The guilt appeal in a study does not recommend any counteracting behaviors or measures to undo the perceiver’s wrongdoing and reduce its harm to victims.

F. **Outcome type** (1 = Guilt; *2 = Knowledge; 3 = Attitude; 4 = Behavioral intention; 5 = Behavior, 6 = Other emotion than guilt; 7 = Motivation; 8 = Cognition; *9 = Efficacy; and *10 = Other).

Operational definition and coding instructions:

The variable records the specific type of outcome variables that are measured in a study.

(1) Guilt: The study measures the emotional feelings of guilt as an outcome.

(2) Knowledge: The study measures participants’ levels of knowledge or their understanding of a particular object.

(3) Attitude: The study measures participants’ opinions or feeling about a persuasion object.

(4) Behavioral intention: The study measures participants’ readiness or willingness to perform a behavior.

(5) Actual Behavior: The study measures whether or not participants have actually performed a behavior.

(6) Emotion other than guilt: The study measures a specific emotional feeling,

excluding guilt, as an outcome, such as joy.

(7) Motivation: The study measures motivation as an intermediate process that directs emotional arousal and cognitive or behavioral activities in response to external stimuli.

(8) Cognition: The study measures a specific cognitive process or outcome related to acquiring and understanding information and knowledge.

(9) Efficacy: A person's perceived ability or power to generate an effect, such as efficacy of conducting cervical cancer screening.

(10) Other: The study measures an outcome that does not belong to any of the categories above.

G. **Context** (1 = Advertising/Marketing; 2 = Education; 3 = Environment; 4 = Medical/Health-related; *5 = Political-related; *6 = Safety instruction; and 7 = Other);

Operational definition and coding instructions:

This variable records the circumstances or situation on which the subject, stimulus event, and purpose of guilt appeal are based. For example, a guilt appeal that advocates recycling behaviors is related an environmental context.

(1) Advertising/Marketing: The guilt appeal in a study is related to selling products or services.

(2) Education: The guilt appeal in a study is related to obtaining knowledge in general or teaching and learning in a school or non-school setting.

(3) Environment: The guilt appeal in a study is related to protecting the natural environment.

(4) Medical/Health-related: The guilt appeal in a study is related to medical services for physical and/or mental wellbeing at a hospital or non-hospital setting.

(5) Political-related: The guilt appeal in a study is related to the government, legislation, or the way a country/region is governed.

(6) Safety instruction: The guilt appeal in a study is related to reducing the risk in daily lives or that of operating machinery.

(7) Other: The guilt appeal in a study is related to a context that is not mentioned above.

H. **Induction Methods** (1 = Persuasive message; 2 = Recall; 3 = Other):

Operational definition and coding instructions:

This variable records the initial processes of eliciting guilt in guilt appeals. Specifically, coders check how the study matches the core appraisal pattern of guilt to arouse the emotion.

(1) Persuasive message: The study provides each participant with a brief piece of information that is produced on a specific medium to match the core appraisal pattern of guilt.

(2) Recall: The study asks each participant to remember an incident in their own lives that once aroused guilt and then to re-experience it again now.

(3) Other: The study uses an induction method that does not belong to the first two categories.

I. **Narrative** (1 = Narrative; 2 = Non-narrative):

Operational definition and coding instructions:

This variable records whether the study arouses guilt by using a story versus by using

reasoning, logical arguments, and statistical information.

(1) Narrative: The study provides stories involving fictional characters and settings to arouse guilt. If the study asks the participants to write or recount their own experiences through storytelling, this option is also coded.

(2) Non-narrative: The study uses reasoning, logical arguments, and statistical information to arouse guilt. If the study asks the participants to write logicl arguments by themselves, this option is also coded.

J. **Modalities** (1 = Text only; 2 = Text and image; *3 = Text and video;-*4 = Image only; *5 = Video only; 6 = Audio only; *7 = Text and audio; 8 = Unspecified).

Operational definition and coding instructions:

This variable records the modality of the stimulus or induction materials used in a study as a guilt appeal. Modality refers to the type of output channel or media through which the guilt appeal information is delivered from the experimenter to the participants as perceivers.

(1) Text only: The study uses written words only in the stimulus material of a guilt appeal.

(2) Text and image: The study uses both written words and static visual elements in the stimulus material of a guilt appeal.

(3) Text and video: The study uses both written words and moving visual elements in the stimulus material of a guilt appeal.

(4) Image only: The study uses static visual elements only in the stimulus material of a guilt appeal.

(5) Video only: The study uses moving visual elements only in the stimulus

material of a guilt appeal.

(6) Audio only: The study uses sound only in the stimulus material of a guilt appeal.

(7) Text and audio: The study uses written words and sound the stimulus material of a guilt appeal.

(8) Unspecified: The study does not use a modality that is listed above. This option also applies to the study that does not provide details regarding the modality of the guilt appeal.

K. **Time Points of measurement** (1= Immediately after/within a day; 2 = Moderate delay/1-7 days; 3 = Long delay/More than 7 days):

Operational definition and coding instructions:

This variable records the time when the study measures outcome variables after presenting guilt appeals. The measurement time points may vary between immediate to several days after guilt appeals occur.

(1) Immediate: The study measures outcome variables immediately or within a day after guilt appeals occur.

(2) Within a week: The study measures outcome variables about 1-7 days after guilt appeals occur.

(3) After a week: The study measures outcome variables more than 7 days after guilt appeals occur.

Note. Categories marked with an asterisk (*) were excluded from data analysis due to zero or very few cases coded.

Appendix B: Calculation of Effect Sizes

Because effect sizes might be biased for small sample sizes, they were first corrected for the small sample bias (Hedges, 1981; Hedges & Olkin, 1985). The unbiased standardized effect size (Hedges' *g*) can be computed from a series of formulas. Given that effect sizes are known to be biased for small sample sizes, Hedges' *g* could adjust the bias via formula $g = d * [1 - 3/4(n_1 + n_2 - 1)]$ (Hedges & Olkin, 1985, page 81). The unbiased effect size (*g*) was used in subsequent analyses.

A positive Hedges' *g* indicated an increase of the effect was elicited by guilt appeals compared to an equivalent non-guilt or true control condition on study outcomes; a negative Hedges' *g* indicated a decrease of the effect was elicited by guilt appeals compared to an equivalent non-guilt or true control condition on outcomes. For studies where the guilt appeal was linked to a reduction in outcome variables for which a higher score indicated negative effects, the computed sign of the effect size was reversed so all positive differences reflected an increase in the dependent variables.

For pre-post and control-treatment (i.e., between within-subject) experimental studies, the standardized mean change for the treatment group and control group (*g*) was computed using formula: $g = [(\bar{Y}_t - \bar{X}_t) - (\bar{Y}_c - \bar{X}_c)] / S_{pooled}$,where $\bar{Y}_t$ and $\bar{Y}_c$ are post-test mean scores of outcome variable and $\bar{X}_t$ and $\bar{X}_c$ are pre-test mean scores of outcome variables; S_{pooled} is the pooled standard deviation of outcome variables, which is

$$S_{pooled} = \sqrt{\frac{(n_t - 1) * SD_{Xt}^{\ 2} + (n_c - 1) * SD_{Xc}^{\ 2} + (n_t - 1) * SD_{Yt}^{\ 2} + (n_c - 1) * SD_{Yc}^{\ 2}}{2(n_t + n_c - 2)}}$$

(Morris, 2008) where n_t and n_c are sample size for treatment and control groups; SD_{Xt} and SD_{Xc} are

standard deviation of the pre-tests for treatment and control groups; SD_{Yt} and SD_{Yc} are standard deviation of the post-tests for treatment and control groups (Morris, 2008).

If no comparison group was used (i.e., within-subject experiment design), the standard deviation mean (*g*) was computed as $g = [(\overline{Y} - \overline{X})] / [S_{pooled} / \sqrt{2(1 - r_{XY})}]$ (Lipsey & Wilson, 2001), where $\overline{X}$ and $\overline{Y}$ are pre-test and post-test means of outcome variable; S_{pooled} is the pooled standard deviation of pre-test and post-test; r_{XY} is the correlation between pre-test and post-test score. In cases where the correlation between pre-test and post-test score was not reported, r_{XY} of 0.5 was used to compute g as a default (Lipsey & Wilson, 2001).

For post-test control group (i.e., between-subject) design, the standardized mean difference (*g*) was computed using the formula $g = [(\overline{Y}_t - \overline{Y}_c)] / S_{pooled}$, where S_{pooled} is the pooled standard deviation of $\overline{Y}_t$ and $\overline{Y}_c$, which is computed as

$$S_{pooled} = \sqrt{\frac{(n_t - 1) * SD_t^{\,2} + (n_c - 1) * SD_c^{\,2}}{n_t + n_c - 2}}$$

,where $\overline{SD}_t$ and $\overline{SD}_c$ are standard deviation and $\overline{Y}_t$ and $\overline{Y}_c$ are outcome for treatment group and control group. In cases where mean and standard deviations of each group were not reported, test statistics such as *F* and *t* values were used to calculate Hedges' *g* (Rosenthal, 1994).

References for Appendices A and B

Hedges, L. V. (1981). Distribution theory for Glass's estimator of effect size and related estimators. *J. Educ. Behav. Stat.* 6, 107–128. doi: 10.3102/10769986006002107.

Hedges, L. V., and Olkin, I. (1985). "Random effects models for effect sizes," in *Statistical methods for meta analysis*, eds. L. V. Hedges and I. Olkin (Academic Press), 189–203.

Lipsey, M. W., and Wilson, D. B. (2001). *Practical meta-analysis*. Thousand Oaks, CA: Sage Publications, Inc.

Morris, S. B. (2008). Estimating effect sizes from pretest-posttest-control group designs. *Organizational Research Methods* 11, 364–386. doi: 10.1177/1094428106291059.

Rosenthal, R. (1994). "Parametric measures of effect size," in *The handbook of research synthesis and meta-analysis*, eds. H. Cooper, L. V. Hedges, and J. C. Valentine (New York, NY: Russell Sage Foundation), 231–244.

Appendix C: References of the articles included in the meta-analysis

*denotes a paper that reports studies with outliers.

Agrawal, N., and Duhachek, A. (2010). Emotional compatibility and the effectiveness of antidrinking messages: A defensive processing perspective on shame and guilt. *J. Mark. Res.* 47, 263–273. doi: 10.1509/jmkr.47.2.263.

*Allard, T., and White, K. (2015). Cross-domain effects of guilt on desire for self-improvement products. *J. Consum. Res.* 42, 401–419. doi: 10.1093/jcr/ucv024.

Allspach, L. E. (2000). The character of guilt within interpersonal relationships: A comparison of friends and significant others. Available at: https://www.proquest.com/dissertations-theses/character-guilt-within-interpersonal/docview/304580337/se-2?accountid=14244.

Antonetti, P., Baines, P., and Jain, S. (2018). The persuasiveness of guilt appeals over time: Pathways to delayed compliance. *J. Bus. Res.* 90, 14–25. doi: 10.1016/j.jbusres.2018.03.030.

Boster, F. J., Mitchell, M. M., Lapinski, M. K., Cooper, H., Orrego, V. O., and Reinke, R. (1999). The impact of guilt and type of compliance-gaining message on compliance. *Commun. Monogr.* 66, 168–177. doi: 10.1080/03637759909376470.

Chang, C.-T. (2011). Guilt appeals in cause-related marketing: The subversive roles of product type and donation magnitude. *International Journal of Advertising* 30, 587–616. doi: 10.2501/ija-30-4-587-616.

Chang, C.-T. (2012). Are guilt appeals a panacea in green advertising? The right formula of issue proximity and environmental consciousness. *International Journal of Advertising* 31, 741–771. doi: 10.2501/IJA-31-4-741-771.

Compton, J., and Pfau, M. (2008). Inoculating against pro-plagiarism justifications: Rational and affective strategies. *J. Appl. Commun. Res.* 36, 98–119. doi: 10.1080/00909880701799329.

Graton, A., Ric, F., and Gonzalez, E. (2016/1). Reparation or reactance? The influence of guilt on reaction to persuasive communication. *J. Exp. Soc. Psychol.* 62, 40–49. doi: 10.1016/j.jesp.2015.09.016.

Jiménez, M., and Yang, K. C. C. (2008). How guilt level affects green advertising effectiveness? *Journal of Creative Communications* 3, 231–254. doi: 10.1177/097325861000300301.

Kjell, O. N. E., and Thompson, S. (2013). Exploring the impact of positive and negative emotions on cooperative behaviour in a Prisoner’s Dilemma Game. *PeerJ* 1, e231. doi: 10.7717/peerj.231.

Lee, Y.-K. (2013). The influence of message appeal, environmental hyperopia, and environmental locus of control on green policy communication. *Social Behavior and Personality: an international journal* 41, 731–738. doi: 10.2224/sbp.2013.41.5.731.

Noble, G., Pomering, A., and Johnson Lester, W. (2014). Gender and message appeal: Their influence in a pro-environmental social advertising context. *Journal of Social Marketing* 4, 4–21. doi: 10.1108/JSOCM-12-2012-0049.

Peloza, J., White, K., and Shang, J. (2013). Good and guilt-free: The role of self-accountability in influencing preferences for products with ethical attributes. *J. Mark.* 77, 104–119. doi: 10.1509/jm.11.0454.

Shehu, E., Langmaack, A.-C., and Clement, M. (2013). The dark side of using emergencies in direct marketing campaigns for blood donation services. *Service Science* 5, 163–178. doi: 10.1287/serv.1120.0041.

Simunich, B. A. (2008). Emotion arousing message forms and personal agency arguments in persuasive messages: Motivating effects on pro-environmental behaviors. Available at: https://rave.ohiolink.edu/etdc/view?acc_num=osu1228334861 [Accessed February 9, 2021].

Steinhauer, M. (2016). Effects of shame-consistent versus guilt-consistent feedback on retailatory and displaced aggression. Available at: https://search.proquest.com/pqdtglobal/docview/1803599064/A2F22794C68A4BC8PQ/1?accountid=14585.

Stoll, B., Edwards, C., and Edwards, A. (2016). "Why aren't you a sassy little thing": The effects of robot-enacted guilt trips on credibility and consensus in a negotiation. *Communication Studies* 67, 530–547. doi: 10.1080/10510974.2016.1215339.

Appendix D: List of Acronyms

**Acronym**	**Full name**
CI	Confidence Interval
H	Hypothesis
ICC	Intraclass Correlation
M	Mean
PICOS	Population, Intervention, Comparator, Outcomes, Study design
PRISMA	Preferred Reporting Items for Systematic Reviews and Meta-Analyses
RQ	Research Question
RVE	Robust Variance Estimation
SD	Standard Deviation
SE	Standard Error
